# Transfer RNA-Derived Fragments, the Underappreciated Regulatory Small RNAs in Microbial Pathogenesis

**DOI:** 10.3389/fmicb.2021.687632

**Published:** 2021-05-17

**Authors:** Zhongyou Li, Bruce A. Stanton

**Affiliations:** Department of Microbiology and Immunology, Geisel School of Medicine at Dartmouth, Hanover, NH, United States

**Keywords:** transfer RNA fragments, regulatory RNA, extracellular vesicles, outer membrane vesicles, microbial small RNAs, host-pathogen interaction

## Abstract

In eukaryotic organisms, transfer RNA (tRNA)-derived fragments have diverse biological functions. Considering the conserved sequences of tRNAs, it is not surprising that endogenous tRNA fragments in bacteria also play important regulatory roles. Recent studies have shown that microbes secrete extracellular vesicles (EVs) containing tRNA fragments and that the EVs deliver tRNA fragments to eukaryotic hosts where they regulate gene expression. Here, we review the literature describing microbial tRNA fragment biogenesis and how the fragments secreted in microbial EVs suppress the host immune response, thereby facilitating chronic infection. Also, we discuss knowledge gaps and research challenges for understanding the pathogenic roles of microbial tRNA fragments in regulating the host response to infection.

## Introduction

To thrive in harsh conditions, micro-organisms must respond to changes in the environment, including iron deprivation, nutrient starvation, and host immune responses. The transcriptional and post-transcriptional regulation by small RNAs (sRNAs) is a highly efficient mechanism to reshape microbial transcriptomes and metabolism in response to changes in the environment. Conventional microbial sRNAs are heterogeneous in size, ranging from 50 to 500 nt, and regulate gene expression by base-pairing with the translation initiation region or coding sequence of target mRNAs or by acting as sRNA sponges (Carrier et al., 2018). In the past decade, microbial non-coding sRNAs (ncRNAs) originating from intergenic regions have been characterized and reviewed extensively (Wagner and Romby, 2015; Carrier et al., 2018). Usually, ncRNAs from the intergenic regions have their promoters, and hence their expression is dictated by promoter activity and transcriptional regulators. Recently, it has become evident that intergenic regions are not the only source of microbial sRNAs. For example, sRNAs cleaved from 5' or 3' untranslated regions of mRNAs with regulatory roles have been reported (Ren et al., 2017; Manna et al., 2018).

Advancements in sRNA sequencing technologies and bioinformatics tools have led to the discovery of transfer RNA (tRNA)-derived fragments, leading to functional studies that have elucidated the regulatory roles of these fragments (Su et al., 2020). In eukaryotes and prokaryotes, tRNAs are the most abundant RNA species by the number of molecules (Neidhardt, 1996; Palazzo and Lee, 2015) and are evolutionarily conserved. Although most studies examining the role of tRNA-derived fragments have been conducted in mammals, in this mini-review, we focus on studies describing the biogenesis and cell-autonomous effects of microbial tRNA fragments. Also, we review papers that report the role of microbial tRNA fragments secreted in extracellular vesicles (EVs) and how they regulate gene expression in the host.

Extracellular vesicles secreted by microbes serve various functions, including intra-kingdom and inter-kingdom transfer of toxins, virulence factors, DNA, sRNAs, and tRNA fragments (Wang and Fu, 2019; Ahmadi Badi et al., 2020). There are several categories of EVs based on their origin, biogenesis, size (10–1,000 nm), and composition (Théry et al., 2018; Toyofuku et al., 2019). One of the main functions of EVs is to protect their RNA content from RNases present in the extracellular environment. In bacteria, the most studied EVs secreted by Gram-negative bacteria are outer membrane vesicles (OMVs) with a single bilayer derived from the outer membrane (Jan, 2017). Also, outer-inner membrane vesicles (O-IMVs), having a double bilayer composed of the outer and inner membranes, are secreted by Gram-negative bacteria, but only represent a very small fraction of EVs (Pérez-Cruz et al., 2015). OMVs and O-IMVs are ~100 nm vesicles that subserve various functions, including pathogen-pathogen interactions, and delivering virulence factors, sRNAs and tRNA fragments into host cells to modulate the host immune response (Kaparakis-Liaskos and Ferrero, 2015; Koeppen et al., 2016; Vitse and Devreese, 2020). Gram-positive bacteria and fungal pathogens also secrete EVs that contain sRNAs, tRNAs, and tRNA fragments (Peres da Silva et al., 2015; Resch et al., 2016; Alves et al., 2019; Rodriguez and Kuehn, 2020; Joshi et al., 2021). In sum, microbial EVs containing sRNAs and tRNA fragments deliver their contents to recipient cells, and a few studies have shown by transfecting synthetic RNA oligos and making sRNA-deletion mutants, that sRNAs and tRNAs regulate gene expression in eukaryotic host cells (Garcia-Silva et al., 2014a; Koeppen et al., 2016; Choi et al., 2017; Zhang et al., 2020).

This article summarizes the current stage of knowledge regarding the biogenesis of microbial tRNA fragments and their regulatory roles in pathogens and host cells. In addition, important knowledge gaps and research challenges regarding their roles in microbial pathogenesis will be discussed.

## Microbial tRNA Fragments and Their Biogenesis

**Figure 1:**
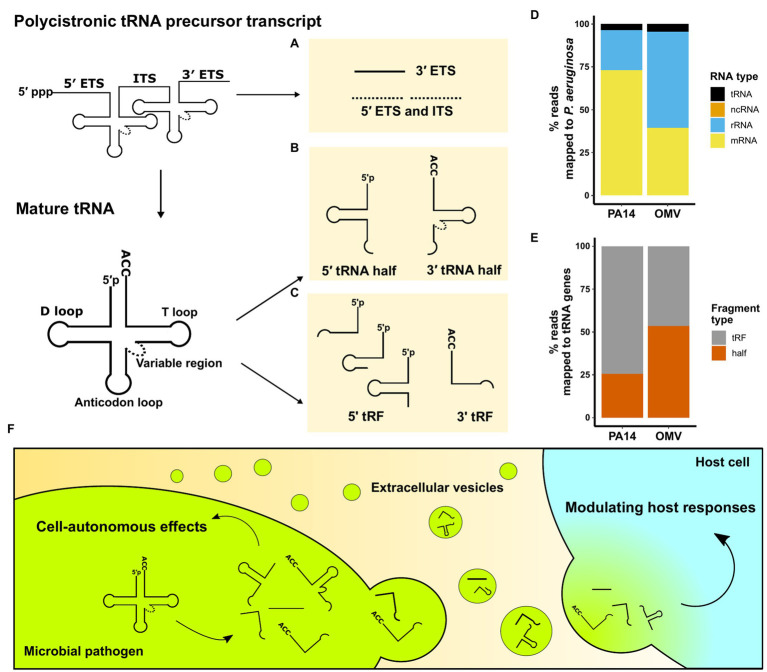
An overview of microbial tRNA-derived fragments. **(A–C)** Three categories of tRNA fragments have been identified based on their origin and processing. Stable tRNA fragments can originate from polycistronic pre-tRNA transcripts or mature tRNAs. **(A)** ETS and internal transcribed spacer (ITS) sequences are excised from the pre-tRNA transcripts; however, no stable 5' ETS and ITS (dotted) have been reported. **(B)** Cleavage at the anticodon loops creates 5' tRNA half and 3' tRNA half fragments. **(C)** RNases cleave near D loops and T loops to generate 5' tRFs and 3' tRFs. **(D)** sRNA unique sequencing reads from *P. aeruginosa* strain PA14 cells and their OMVs. Non-coding sRNAs (ncRNAs) are not visible on the plot as they accounted for less than 0.02% of total reads. **(E)** tRNA fragment type distribution of unique reads mapped to PA14 tRNA genes in PA14 cells and OMVs. Sequence reads in **(D)** and **(E)** are from Koeppen et al. (2016). **(F)** Schematic representation of microbial tRNA fragment-mediated effects in pathogens and the pathogen-to-host interaction *via* EVs.

Transfer RNAs are 70–100 nucleotides (nt) long molecules with highly conserved sequences that form secondary cloverleaf and L-shaped three-dimensional structures (Fujishima and Kanai, 2014). Besides their well-known function as amino acid carriers to decode mRNA sequences in eukaryotes, numerous non-canonical roles of tRNAs have been identified, including tRNA fragment mediated gene silencing through an Argonaute-microRNA like mechanism (Maute et al., 2013) and both negative and positive effects on global regulation of protein translation (Yamasaki et al., 2009; Kim et al., 2017). The roles of tRNA fragments in eukaryotes have been extensively reviewed (Keam and Hutvagner, 2015; Su et al., 2020). tRNA fragments were first described in *Escherichia coli* in response to bacteriophage infection (Levitz et al., 1990). Over the past decades, using northern blot analysis and sRNA sequencing techniques, tRNA fragments have been found in all domains of life and are not random degradation products (Kumar et al., 2014). This article summarizes identified microbial tRNA fragments into three categories (Figures 1A–C). They are either derived from a polycistronic tRNA precursor transcript or a mature tRNA and include: (1) external transcribed spacer (ETS) and internal transcribed spacer (ITS) sequences, (2) 5' and 3' tRNA halves, and (3) 5' tRNA-derived fragments (5' tRFs) and 3' tRNA-derived fragments (3' tRFs).

The first category of tRNA fragments is the spacer RNAs arising from the tRNA maturation process (Figure 1A). tRNA precursors contain a 5' ETS and a 3' ETS. Prokaryotic tRNA precursors from operons have ITS to separate tRNAs. Multiple ribonucleases, including RNase P and E, are required to generate mature tRNAs and release ETS and ITS (Esakova and Krasilnikov, 2010; Shepherd and Ibba, 2015).

The second category of tRNA fragments consists of 5' tRNA halves and 3' tRNA halves, which are products of endoribonuclease-mediated cleavage in the anticodon loop of mature tRNAs (Figure 1B). 5' tRNA halves are 32–40 nucleotides from the 5′ terminus of mature tRNAs, while 3' halves are 40–50 bases from the anticodon loop to the 3' terminus due to the variable region and 3'-CCA. Various anticodon ribonucleases (ACNases) targeting the anticodon loop have been identified. In *E. coli*, T4 phage infection activates PrrC, a tRNA^Lys^-specific ACNase, as one of the suicidal events to protect the population from infection (Morad et al., 1993). Colicin E5 and Colicin D secreted from *E. coli* are ACNases that cleave multiple tRNAs of competing *E. coli* strains in half (Ogawa et al., 1999; Tomita et al., 2000). Ogawa et al. (2020) revealed that the anticodon-loop sequence of tRNA^Arg^ determines its susceptibility to colicin D, and they also demonstrated that the cleaved tRNA^Arg^ occupies the ribosome A-site leading to the impairment of translation. Similarly, fungi secrete different ribotoxins that generate 5' tRNA halves and 3' tRNA halves which inhibit cell growth of nonself yeast cells (Lu et al., 2005; Chakravarty et al., 2014). *Mycobacterium tuberculosis* produces MazF-mt9 that cleaves tRNA^Lys^ in half, which downregulates bacterial growth (Schifano et al., 2016). Ribotoxins including VapCs in *M. tuberculosis*, *Shigella flexneri*, and *Salmonella enterica* target the anticodon loop of different tRNAs to modulate translation (Winther and Gerdes, 2011; Winther et al., 2016).

The third category of tRNA fragments includes 5' tRFs and 3' tRFs. These tRFs are cleavage products near the D loop or in the T loop of mature tRNAs (Figure 1C). 5' tRFs start from the extreme 5' end into the D stem, D loop, or anticodon stem; hence, 5' tRFs usually range from 18 to 31 nt. 3' tRFs are defined as sRNAs derived from cleavage in the T loop with a 3'-CCA end and about 18–22 nt in length. MazF-mt9 toxin in *M. tuberculosis* has been reported to cleave tRNA^Pro^ to generate 5' tRFs (Schifano et al., 2016). Due to the size similarity between microRNAs (miRNAs) in eukaryotes and tRFs, the miRNA generation machinery has been implicated in generating 5' tRFs and 3′ tRFs. Dicer, which plays a major role in the maturation of miRNAs, also cleaves several mature tRNAs to generate tRFs in vertebrates (Cole et al., 2009; Shigematsu and Kirino, 2015). sRNA sequencing analyses comparing wildtype and Dicer knockout cells in different eukaryotes reveal that Dicer is dispensable for the biogenesis of the majority of 5' tRFs and 3' tRFs (Li et al., 2012; Kumar et al., 2014); however, in prokaryotes, the biogenesis of tRFs remains largely unknown at this time.

## Microbial tRNA Fragments and Their Cell-Autonomous Effects

Lalaouna et al. (2015) identified the first functional tRNA fragment from an ETS in *E. coli* and demonstrated that the 3' ETS of *leuZ* (3' ETS*^leuz^*) is an sRNA sponge that inhibits the activity of target sRNAs, RyhB, and RybB. RyhB and RybB regulate iron homeostasis and outer membrane integrity, respectively. To demonstrate the physiological role of 3' ETS*^leuZ^* in *E. coli*, the authors reported that it prevents cells from using succinate as the sole carbon source and decreases antibiotic colicin sensitivity. Importantly, they demonstrated that Hfq, a conserved bacterial RNA-binding protein, is required to form the tRNA fragment-sRNA target complex.

Gebetsberger et al. (2012) sequenced sRNAs co-purified with ribosomes of *Haloferax volcanii*, a halophilic archaeon, and identified multiple 5' tRFs. They found that cells grown under elevated pH have abundant 5' tRNA^Val^ fragment, which binds to small ribosomal subunits to inhibit translation globally. In addition, the 5' tRNA^Val^ fragment of *H. volcanii* also attenuates protein synthesis by *S. cerevisiae* and *E. coli*’s ribosomes (Gebetsberger et al., 2017). Also the 3' tRNA^Thr^ half from *Trypanosoma brucei* has a similar effect in *H. volcanii* and *S. cerevisiae* to mediate translation stimulation (Fricker et al., 2019). These reports suggest a functionally conserved mode of action across life domains. Similar regulatory roles of 5' tRFs and 3' tRFs in *S. cerevisiae* have also been identified. Several tRFs in yeast bind to the small ribosomal subunits and aminoacyl-tRNA synthetases to reduce global translation (Mleczko et al., 2018).

Although 5' and 3' tRNA halves have been identified in many studies, the cell-autonomous effects of tRNA halves are largely unknown. Studies in *M. tuberculosis* and *Aspergillus fumigatus* suggest that micro-organisms maintain their persistence in host cells or remain in a life stage by cleaving tRNAs in half (Jöchl et al., 2008; Winther et al., 2016). Selective human 5' tRNA halves identified in saliva have high sequence similarity to tRNAs of *Fusobacterium nucleatum*, an oral opportunistic pathogen. Co-culture of human 5' tRNA halves with *F. nucleatum* inhibits bacterial growth, likely through interference with bacterial protein biosynthesis (He et al., 2018). Several studies have also demonstrated that environmental stress increases cytosolic tRNA halves in microbes (Thompson et al., 2008; Garcia-Silva et al., 2010; Fricker et al., 2019; Raad et al., 2021); however, the function of 5' and 3' tRNA halves induced by stress remains elusive.

Taken together, these initial reports describing the ubiquity and the function of cytosolic tRNA fragments in microbial cells and the differential expression of specific tRNA fragments (summarized in Table 1) suggest their possible roles in bacterial homeostasis and in regulating the expression of virulence factors.

**Table 1:** Studies identifying and characterizing microbial transfer RNA (tRNA) fragments.

Microbial species	Description of identified tRNA fragments	Proposed function and relevant findings of tRNA fragments	Reference
*Aspergillus fumigatus* (ATCC46645)	5' and 3' tRNA halves from multiple tRNAs during conidiogenesis were detected by northern blot analysis.	The cleavage of tRNAs into halves causes conidial tRNA depletion leading to the resting stage of *A. fumigatus*	Jöchl et al., 2008
*Saccharomyces cerevisiae*	5' and 3' tRNA halves from multiple tRNAs were detected by northern blot analysis during oxidative stress and stationary phase.	tRNA cleavage is not a function of quality control and unlikely to inhibit protein synthesis.	Thompson et al., 2008; Thompson and Parker, 2009
*Trypanosoma cruzi*	About 25% of small RNAs (sRNAs) sequenced in the unicellular parasites were 5' tRNA halves from three specific tRNA isoacceptors.	Nutritional stress induces a significant increase of tRNA halves.	Garcia-Silva et al., 2010
*Trypanosoma cruzi*	tRNA fragments were the second most abundant class in extracellular vesicles (EVs). A high level of tRNA halves from specific tRNA isoacceptors suggests differential packaging.	5' tRNA halves are transferred between parasites to host cells *via* EVs. 5' tRNA halves affect gene expression patterns in host cells.	Bayer-Santos et al., 2014; Garcia-Silva et al., 2014a,b
*Escherichia coli* (MG1655)	Abundant tRNA fragments were found in both *E. coli* cells and outer membrane vesicles (OMVs). Over 90% of sRNA reads in OMVs were mapped to tRNA fragments.	The first report providing detailed profiling of the RNA content in *E. coli* cells and their OMVs and suggesting selective tRFs are packed into EVs.	Ghosal et al., 2015
*Escherichia coli* (MG1655)	Excised 3' external transcribed spacer (ETS) of *leuZ* (3' ETS*^leuZ^*, 53 nt-long) was co-purified with sRNAs RyhB, and RybB.	3' ETS*^leuZ^* is excised from the pre-tRNA transcript and acts as an sRNA sponge in *E. coli* to suppress the activity of sRNAs modulating tricarboxylic acid cycle fluxes and antibiotic sensitivity.	Lalaouna et al., 2015, 2017
*Candida albicans*, *Cryptococcus neoformans*, *Paracoccidioides brasiliensis*, *Saccharomyces cerevisiae*	A high abundance of 3' tRF Arginine (CCU) was found in fungal EVs. sRNA reads mapped to tRNAs account for 20–60% of all reads.	Arginine tRNA (CCU) is required to synthesize the heat shock protein at high temperatures through the regulation of translational frameshift. The production of 3' tRF from this tRNA is suggested to affect fungal gene expression.	Peres da Silva et al., 2015
*Leishmania donovani*, *Leishmania braziliensis*	About 25–45% of all reads in the *Leishmania* EVs were mapped to tRNA fragments. Among those, 5' tRNA halves from a small subset of the same tRNA isoacceptors accounted for the majority of reads.	tRNA fragments and other sRNAs in EVs are delivered into macrophages.	Lambertz et al., 2015
*Pseudomonas aeruginosa*	Multiple tRNA fragments were detected in microbes and OMVs. sRNA52320, a 5' tRNA^fMet^ fragment and a 5' tRNA^fMet^ half were differentially enriched in the OMVs compared to the cells.	A 5' tRNA^fMet^ fragment is transferred into host cells where it targets the LPS-induced MAPK pathway to reduce IL-8 secretion.	Koeppen et al., 2016
*Escherichia coli* (UPEC) strain 536	In EVs, 27% of sRNA reads were mapped to tRNA fragments, and only 0.23% reads were mapped to mature intact tRNAs.	Detailed profiling of total RNA in EVs secreted by uropathogenic *E. coli* adds to the growing evidence of host-pathogen interactions mediated by sRNAs in EVs.	Blenkiron et al., 2016
*Mycobacterium tuberculosis*	Multiple tRNA halves were identified by using deep sequencing and northern blot analyses.	Twelve anticodon nucleases cleave essential tRNAs to halt protein synthesis leading to survival in host cells.	Winther et al., 2016
*Mycobacterium tuberculosis*	MazF-mt9 toxin cleaves tRNA^Pro14^ and tRNA^Lys43^ in the D loop and anticodon loop, respectively.	The cleavage of specific tRNAs arrests translation and bacterial growth.	Schifano et al., 2016
*Haloferax volcanii*	Multiple 5' tRNA-derived fragments (tRFs) were co-purified with ribosomes and detected by sRNA sequencing and northern blot analyses.	5' tRNA^Val^ fragment production is induced at elevated pH, and the fragment binds to small ribosomal subunits to attenuate global translation.	Gebetsberger et al., 2012, 2017
*Saccharomyces cerevisiae*	Multiple 5' tRFs and 3' tRFs were co-purified with ribosomes and detected by northern blot analyses.	tRFs binds to small ribosomal subunit and aminoacyl-tRNA synthetases to attenuate global translation.	Mleczko et al., 2018
*Trypanosoma brucei*	tRNA halves were significantly induced during nutrient deprivation. 3' tRNA^Thr^ half was the most abundant sRNAs.	3' tRNA^Thr^ half associates with ribosomes to stimulate translation during the recovery from nutritional stress.	Fricker et al., 2019
*Bradyrhizobium japonicum* (rhizobium)	Rhizobial tRFs were identified in soybean nodules.	Rhizobial 5' and 3' tRFs are positive regulators of plant nodulation mediated by AGO1.	Ren et al., 2019
*Escherichia coli*	The susceptibility of four *E. coli* tRNA^Arg^ isoacceptors to colicin D, an anticodon nuclease, was tested.	Cleaved tRNA^Arg^ occupies ribosomal A-site and thus impairs protein translation.	Ogawa et al., 2020
*Helicobacter pylori J99*	3' tRNA^Asn^ half and 5' tRNA^fMet^ fragment were identified as the most abundant and differentially packed sRNAs in EVs.	sR-2509025, a 5' tRNA^fMet^ fragment, is transferred from OMVs into human gastric adenocarcinoma cells and reduced the OMV-induced IL-8 secretion.	Zhang et al., 2020
*Trichomonas vaginalis*	Around 88.2% of sRNA reads in EVs were mapped to tRNA fragments.	5' tRNA halves are the most abundant sRNAs in EVs. The delivery of RNA cargo from EVs to host cells is *via* lipid raft-dependent endocytosis.	Artuyants et al., 2020
*Escherichia coli* (MG1655)	Multiple tRNA fragments were detected in total RNA of cells from different growth stages.	Multiple tRFs are enriched in the stationary but not exponential phase suggesting the possible role of maintaining the stationary phase as well as the bacterial stress response.	Raad et al., 2021

## Extracellular Vesicles are Important Mediators of Intercellular Communication and Delivery of tRNA Fragments to the Host

Intercellular communication mediated by EVs is an important aspect of host-pathogen interaction without direct cell-cell contact (Yáñez-Mó et al., 2015). Secretion of EVs as a mechanism of inter-kingdom and intra-kingdom communication is evolutionarily conserved, as organisms from prokaryotes, plants, and animal cells release EVs into the surrounding environment (Deatherage and Cookson, 2012; Gill et al., 2019; Woith et al., 2019). Identification of regulatory tRNA fragments in EVs has recently garnered attention and led to extensive sRNA content profiling of EVs secreted by Gram-negative and Gram-positive bacteria, fungi, and intracellular parasites (summarized in Table 1). Ghosal et al. (2015) performed the first comprehensive sRNA profiling comparing *E. coli* and their secreted OMVs. In *E. coli* OMVs, over 90% of sequence reads were mapped to tRNA fragments, and the authors reported differential packaging of tRNA fragments in OMVs. In a study sequencing sRNA in *Pseudomonas aeruginosa* (Koeppen et al., 2016), we found that tRNA fragments are more abundant than canonical ncRNAs (Figure 1D), and those tRNA fragments are differentially packaged in OMVs (Figure 1E). Moreover, we exposed human primary bronchial epithelial (HBE) cells to OMVs and used sRNA sequencing to demonstrate that tRNA fragments are the main *P. aeruginosa* sRNAs transferred from OMVs to host cells after exposure. Although tRNA fragments are only a small fraction of sRNAs in OMVs, their predominance in host cells suggests that tRNA fragments may have longer half-lives than other more abundant sRNAs. Moreover, EVs produced by *Trichomonas vaginalis* encapsulate abundant 5' tRNA halves and fuse with benign prostate hyperplasia (BPH-1) cells to deliver sRNAs (Artuyants et al., 2020). In addition, EVs secreted by Gram-positive bacteria, fungi, and even intracellular parasites also deliver protein toxins and sRNA products to host cells to modulate the host immune response (Rodrigues et al., 2008; Rivera et al., 2010; Silverman et al., 2010; Resch et al., 2016; Munhoz da Rocha et al., 2020).

Several studies have reported that tRNA fragments secreted in OMVs are involved in host-pathogen interactions. We demonstrated that sRNA52320, a 24-nt long 5' tRNA^fMet^ fragment, is secreted by *P. aeruginosa* in OMVs that fuse with primary HBE cells and attenuates OMV-induced IL-8 secretion and recruitment of neutrophils into mouse lung by reducing the expression of mitogen-activated protein kinases (MAPK; Koeppen et al., 2016). In our study, we used miRanda, a miRNA target prediction algorithm, to reveal that the 5' tRNA^fMet^ fragment has specific targets in the MAPK signaling pathway in the host, including MAP2K2, MAP2K3, MAP2K4, MAP3K7, and PIK3R2. An unbiased proteomics approach confirmed our target predictions. In addition, sR-2509025, a 31-nt 5' tRNA^fMet^ fragment, is secreted by *Helicobacter pylori* in OMVs and is delivered to human gastric adenocarcinoma cells and diminish LPS-induced IL-8 secretion (Zhang et al., 2020). Intriguingly, the 5' tRNA^fMet^ fragments identified from these two independent studies have a high sequence similarity, with only four nucleotides difference in the 24-nt long overlapping region. Moreover, Garcia-Silva et al. (2014a,b) demonstrated that tRNA halves in *Trypanosoma cruzi* EVs are delivered into HeLa cells where the 5' tRNA^Thr^ half modulates the immune response, including upregulating CXCL2 gene expression.

Considering the findings discussed above, we propose that tRNA fragments delivered by microbial EVs to host cells are a widespread mechanism utilized to hijack the host immune response to enhance survival (Figure 1F). Additional studies are required to test this hypothesis, including studies on how microbes regulate the biogenesis of tRNA fragments in response to different environmental stimuli, the molecular mechanisms of differential packaging of tRNA fragments in EVs, and the molecular mechanisms whereby microbial tRNA fragments regulate the eukaryotic host response to infection. It will be informative to determine if microbial tRNA fragments delivered to eukaryotic hosts utilize the same mechanisms that mammalian tRNA fragments utilize (Chen and Shen, 2020).

## Conclusion and Perspectives

Recent studies have demonstrated that tRNA fragments secreted by microbes in EVs regulate gene expression in various hosts, thus representing an important but underappreciated mechanism of host-pathogen interactions. Although progress has been made in elucidating the role of tRNA fragments in regulating gene expression in microbes and their hosts, there is much to be learned. For example, although sRNA sequencing has identified tRNA fragments in microbes and EVs, it is important to note current sequencing limitations and biases. First, most of the current approaches of preparing sRNA sequencing libraries rely on adaptor ligation, which requires 5' monophosphate and 3' OH of RNA molecules. This requirement limits the tRNA fragments that can be identified. For example, 5' ETS with 5' triphosphates and tRNA halves with 2-3-cyclic phosphate at the 3' end or 5' OH created by ACNases are not amenable to adaptor ligation. However, there are methods that use different enzymes to treat RNA samples before adaptor ligation to enrich RNA molecules with different end chemistries (Thomason et al., 2015; Shigematsu et al., 2018). Second, there are more than 90 post-transcriptional modifications found in tRNAs, which are required for their activity, and some of them impede reverse transcription during library preparation and RT-PCR experiments, which could lead to inaccurate profiling of tRNA fragments (Kellner et al., 2010; Lorenz et al., 2017). Also, the stable structure of tRNAs interferes with cDNA synthesis (Zheng et al., 2015; Behrens et al., 2021). Thus, new approaches are needed to identify tRNA fragments more thoroughly. In addition, better algorithms are needed to predict targets of tRNA fragments. Although tRNA fragments regulate gene expression by base-pairing with target genes in host cells, which is reminiscent of microRNA-mRNA interactions in eukaryotes, targeting algorithms for tRNA fragments should be improved due to a variety of factors. For example, sRNAs base-pair to targets with limited complementarity and conserved sequences called seed regions; however, the seed regions of tRFs and tRNA halves have not been fully characterized; thus, a better understanding of seed regions in tRNA fragments is required for a more accurate target prediction. With a better understanding of the targeting rules of tRNA fragments, it would be possible to generalize experiment findings on one tRNA fragment to other pathogens, given the highly conserved tRNA sequences in microbes (Saks and Conery, 2007).

Additional outstanding questions regarding the role of tRNA fragments in mediating host-pathogen interactions are worthy of note. Is the production and packaging of tRNA fragments into microbial EVs regulated? Does the nucleotide sequence or secondary structure determine differential packaging? How is the secretion of EVs regulated? How do microbial tRNA fragments regulate gene expression in eukaryotic cells? To what extent do microbial EVs in plasma regulate multiple organs? Do differences in EV isolation methods affect EV cargo and the effect of EVs on host cell biology? In this regard, the International Society of Extracellular Vesicles (ISEV) has published a suggested set of standards to increase rigor and reproducibility in EV research (Théry et al., 2018).

In conclusion, despite numerous publications demonstrating the existence of tRNA fragments in microbes and in secreted EVs, many details on the immunoregulatory role of tRNA fragments in regulating the host are unknown. We postulate that tRNA fragments are underappreciated molecules in microbial physiology and host-pathogen interactions (Figure 1F). We anticipate that a greater focus on these molecules in future investigations will lead to a more complete understanding of their roles in mediating host-pathogen interactions.

## Author Contributions

ZL and BS conceived and contributed to the writing of the manuscript. All authors contributed to the article and approved the submitted version.

### Conflict of Interest

The authors declare that the research was conducted in the absence of any commercial or financial relationships that could be construed as a potential conflict of interest.
